# Inhibiting *P. fluorescens* biofilms with fluoropolymer-embedded silver nanoparticles: an *in-situ* spectroscopic study

**DOI:** 10.1038/s41598-017-12088-x

**Published:** 2017-09-19

**Authors:** M. C. Sportelli, E. Tütüncü, R. A. Picca, M. Valentini, A. Valentini, C. Kranz, B. Mizaikoff, H. Barth, N. Cioffi

**Affiliations:** 1 Dipartimento di Chimica, Università degli Studi di Bari “Aldo Moro”, V. Orabona, 4, 70126 Bari, Italy; 2 Institute of Analytical and Bioanalytical Chemistry, Ulm University, Albert Einstein Allee, 11, 89081 Ulm, Germany; 3 Dipartimento di Fisica, Università degli Studi di Bari “Aldo Moro”, V. Orabona, 4, 70126 Bari, Italy; 4 Institute of Pharmacology and Toxicology, Ulm University, Medical center, Albert-Einstein-Allee 11, 89081 Ulm, Germany

## Abstract

Surface colonization by microorganisms leads to the formation of biofilms, i.e. aggregates of bacteria embedded within a matrix of extracellular polymeric substance. This promotes adhesion to the surface and protects bacterial community, providing an antimicrobial-resistant environment. The inhibition of biofilm growth is a crucial issue for preventing bacterial infections. Inorganic nanoparticle/Teflon-like (CF_x_) composites deposited via ion beam sputtering demonstrated very efficient antimicrobial activity. In this study, we developed Ag-CF_x_ thin films with tuneable metal loadings and exceptional in-plane morphological and chemical homogeneity. Ag-CF_x_ antimicrobial activity was studied via mid-infrared attenuated total reflection spectroscopy utilizing specifically adapted multi-reflection waveguides. Biofilm was sampled by carefully depositing the Ag-CF_x_ film on IR inactive regions of the waveguide. Real-time infrared spectroscopy was used to monitor *Pseudomonas fluorescens* biofilm growth inhibition induced by the bioactive silver ions released from the nanoantimicrobial coating. Few hours of Ag-CF_x_ action were sufficient to affect significantly biofilm growth. These findings were corroborated by atomic force microscopy (AFM) studies on living bacteria exposed to the same nanoantimicrobial. Morphological analyses showed a severe bacterial stress, leading to membrane leakage/collapse or to extended cell lysis as a function of incubation time.

## Introduction

Silver nanoparticles (AgNPs) are the most widespread used inorganic nanoantimicrobial agent^1^, and are already extensively used in health industry, for food storage, in manufacturing industry, and numerous environmental applications^2^. About two thousand scientific articles have been published in the past decade in peer-reviewed journals relevant to antimicrobial AgNPs, along with over six hundred patents worldwide^3^. Fluoropolymers offer a variety of applications^4^, due to their low chemical reactivity, high melting point, resistance to corrosive environments and high surface resistivity^5^. Moreover, as hydrophobic materials, fluorinated polymers are self-cleaning and anti-sticking^5^. Among these, metal- and metal oxide-Teflon-like (CF_x_) composites have risen high scientific and industrial interest due to their wide range of technological applications in fields such as optoelectronics^6^, photovoltaics^7,8^, medical devices^9^, anti-stain and water repellent coatings^10,11^, gas sensors^12,13^, nanoantimicrobials^14,15^. Nanostructured composites have been successfully fabricated employing technologies such as plasma deposition^16,17^, co-evaporation^18,19^, RF magnetron sputtering^20–22^, physical vapour deposition (PVD)^23^, hybrid sputtering-vapour methods^24,25^, vacuum gas-jet deposition^26^, and ion beam sputtering (IBS)^10^. The combination of Ag nanoantimicrobials and fluoropolymers is still a topic explored only by few research groups^6,8,10,11^. Among all different approaches proposed for the deposition of Ag-fluoropolymer nanocomposites, PVD process (based on the co-evaporation of silver and fluorinated polymers from independent sources) is one of the most diffused, being fully elucidated in several recent papers by the groups of Eilers^19^, and Faupel^18^. In earlier years, Faupel *et al*. also studied magnetron sputtering^22,27^, and, more recently, the combination of evaporation and magnetron sputtering techniques^25^. Other alternative methods consisting of vacuum gas-jet deposition^26^, deposition from active gas phases formed by electron-beams^28^, and non-thermal plasma methods^29^, have been sporadically explored for the deposition of Ag-CF_x_ materials. In the present study, we exploited an ion beam co-sputtering technique for the controlled deposition of Ag-Teflon-like composite materials for the first time. IBS is particularly interesting as a cost-effective and environmentally friendly technology, allowing a high degree of control on processing parameters such as the film thickness and the metal/metal oxide loading^14^. Different metal loadings (φ) and fluoropolymer composition were obtained by independently tuning sputtering conditions of polytetrafluoroethylene (PTFE) and Ag targets. Exceptional in-plane homogeneity was achieved in both chemical composition and morphology. Antibacterial Ag nanophases were proven to be free of potentially toxic inorganic fluorides, which is counterintuitive and different from what was found in homologous systems^14^, thus supporting the utility of sputter-deposited Ag-CF_x_ materials for real-life applications^30^. These materials provide the advantageous characteristics of the fluoropolymer matrix, i.e. anti-stain, anti-fouling, and water repellent; moreover, due to the presence of AgNPs, they can exert a strong and wide antimicrobial activity and –in case of high metal loading– an additional antistatic function. Although the antimicrobial function of AgNPs is mainly related to ion release in solution^31^, to date there is no univocal interpretation of their biocidal mechanism. Several techniques have been used to study AgNP antimicrobial activity, such as microbiological assays^32,33^, scanning electron microscopy (SEM)^32^, transmission electron microscopy (TEM)^34^, surface-enhanced Raman spectroscopy (SERS)^35^, only to cite a few. Infrared - attenuated total reflectance (IR-ATR) spectroscopy is a powerful tool for assessing antimicrobial activity in real time and for studying living bacteria^36^. IR-ATR enables studying entire bacterial cells, cell colonies and biofilms, thereby eliminating artefacts that may arise during the processes required to isolate specific cellular components^37^. If integrated with a flow-through cell, this technique allows for the real-time monitoring of biofilm growth^38,39^. Atomic force microscopy (AFM) is a complementary tool to investigate antimicrobial mechanism by revealing morphology transformation of stressed bacteria^40^. AFM provides high-resolution topographical imaging of biological samples, and has been widely used to study the mechanisms of action of antimicrobial particles and molecules on bacteria^41–43^. Given the intrinsic complexity of the Ag-CF_x_ system and its interaction, within this study, both techniques –flow-through IR-ATR and AFM measurements– have been applied, providing complimentary data on the model bacteria *Pseudomonas fluorescens*. To the best of our knowledge, this is the first example of real-time monitoring of biofilm/nanoantimicrobial interaction using an ATR waveguide modified with an antimicrobial coating. Novel Ag-CF_x_ materials were synthesized and characterized by TEM and AFM, for investigating the inorganic nanophase dispersion within the polymeric matrix and the surface morphology of the obtained coatings. It is known that surface morphology plays an essential role in biofilm formation. X-ray photoelectron spectroscopy (XPS) was used to assess quantitatively the materials surface chemical composition. Antimicrobial ion release kinetics were studied by electro-thermal atomic absorption spectroscopy (ETAAS), while TEM was employed to rule out that entire AgNPs were released, which may represent a (nano)toxicological risk. Finally, for understanding biofilm formation in molecular detail, *in-situ* IR-ATR spectroscopy was applied^44,45^. Ag-CF_x_ antimicrobial properties on *P. fluorescens* living cells were furthermore evaluated via detailed morphological AFM and SEM studies. The combined spectroscopic/morphological approach herein presented for the study of nanomaterial-biofilm interactions has great prospective importance, since it can decouple ion-mediated effects from nanoparticle-mediated and direct contact ones, thus contributing to separately elucidate bioactivity mechanisms such as those specifically based on ionic release.

## Results

### Thin film deposition and characterization

#### IBS deposition, materials morphology, and water/oil repellent properties

**Figure 1:**
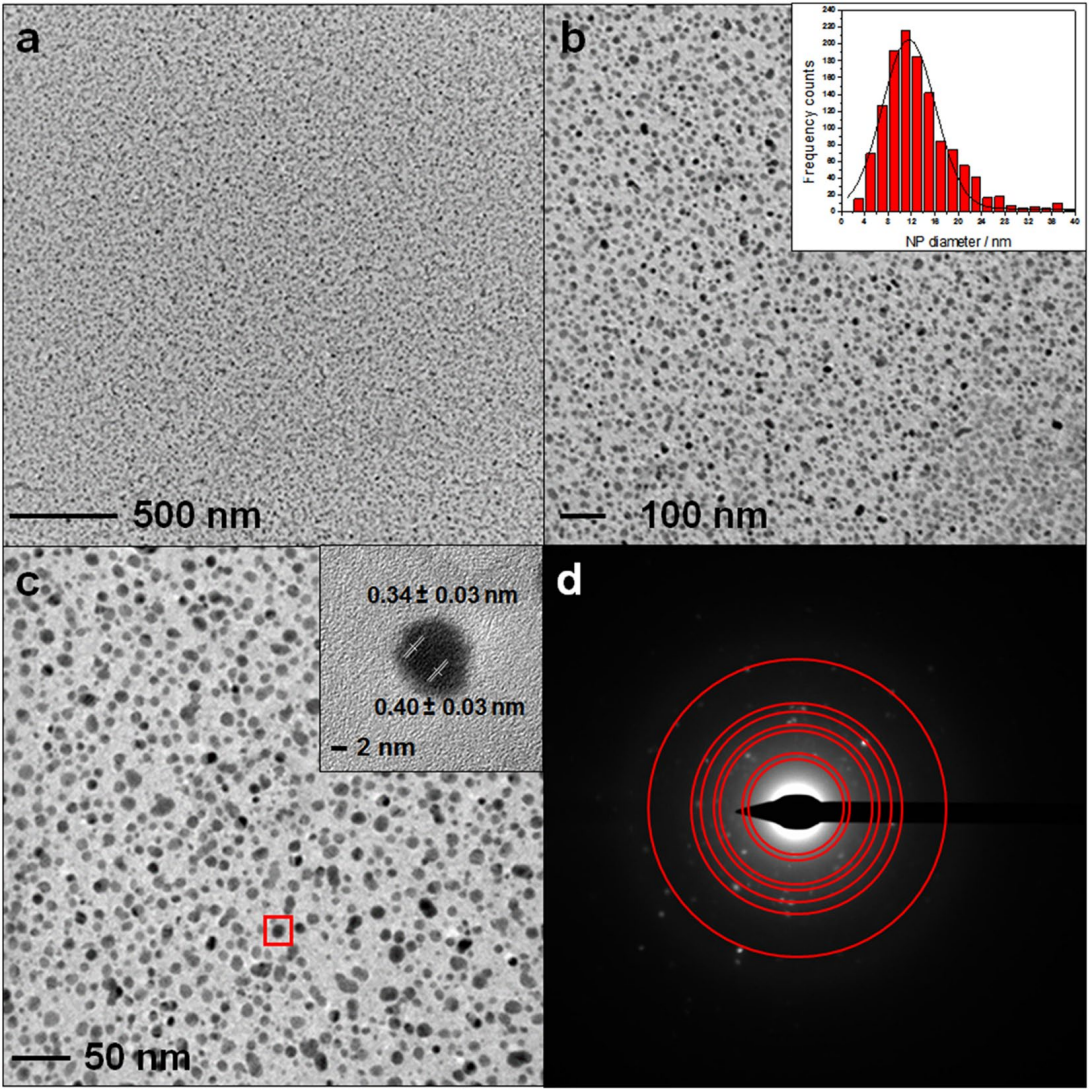
Typical TEM images at increasing magnification of Ag-CF_x_ films with a φ value of 0.25 (**a**,**b**,**c**), along with selected area electron diffraction (SAED) pattern (**d**). Corresponding AgNP size distribution histogram is reported as inset in (**b**). Red square in (**c**) highlights the silver cluster used for interplanar distance measurements (inset in (**c**)).

Ag-CF_x_ nanocomposites with different φ values (0.05 ≤ φ ≤ 0.30) were deposited as 150-nm-thick films. Typical TEM images of Ag-CF_x_ films with a φ value of 0.25 are shown in Fig. 1, along with the AgNPs size distribution. Inorganic nanophases have an average diameter of 9.0 ± 0.3 nm. Some AgNPs showed resolved lattice spacing (inset in Fig. 1c). Two different values of fringe spacing were measurable: 0.34 ± 0.03 nm, corresponding to (111) Ag_2_O interplanar distance, and 0.40 ± 0.03 nm, attributable to (111) Ag^0^ one^46^. In Fig. 1d, different rings were identified, as a superposition of Ag^0^ and Ag_2_O reflections^47^; from inner to outer, they were attributed to: Ag_2_O (111), Ag (111), Ag_2_O (220), Ag (220), Ag_2_O (222), Ag (222), and Ag (420)^48^.

**Figure 2:**
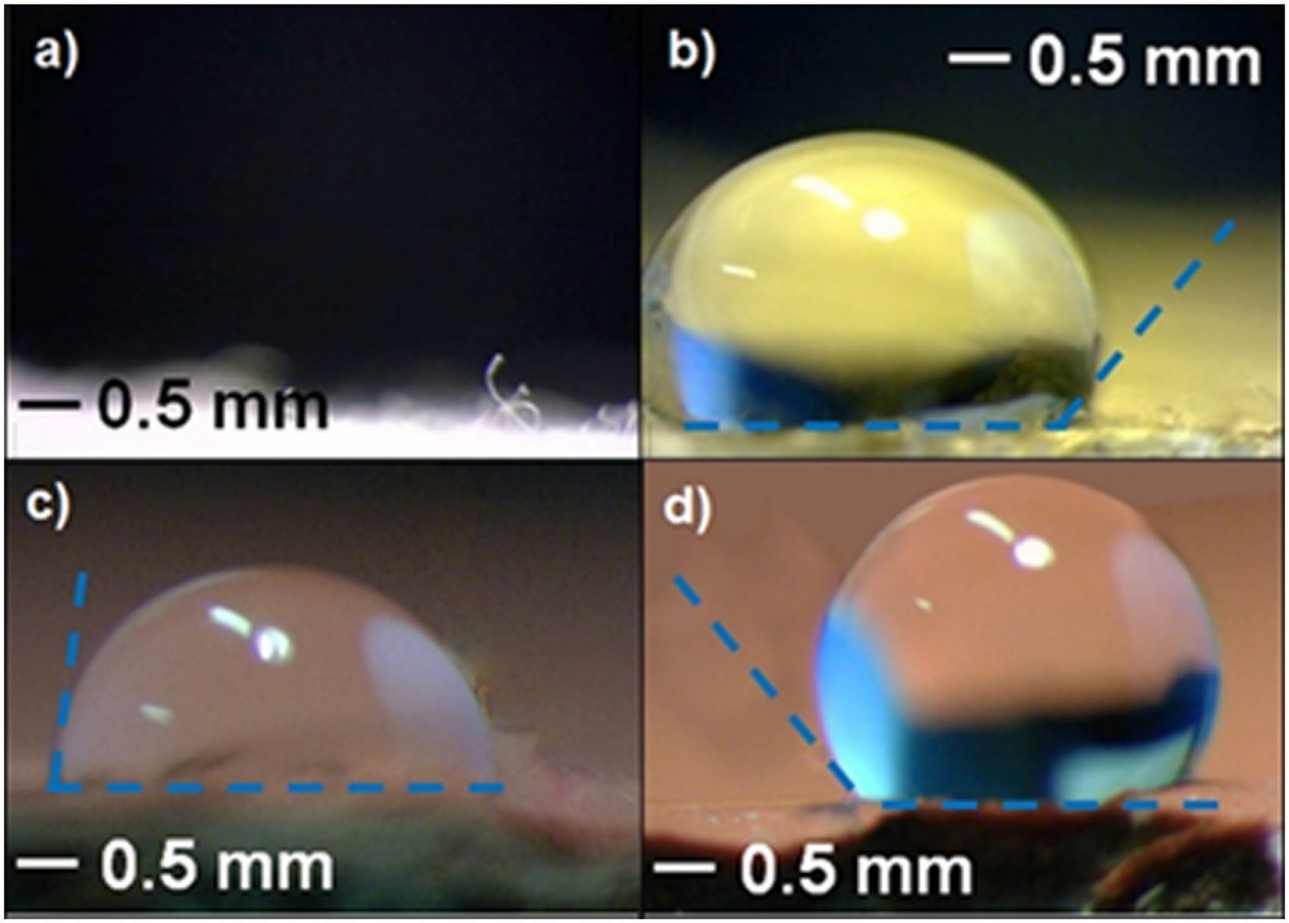
Oil contact angle measurements on: (**a**) untreated textile, where the droplet is completely absorbed by the fabric; (**b**) textile modified by an Ag-CF_x_ composite film with φ = 0.15. Water contact angle measurements on: (**c**) untreated leather; (**d**) leather modified by an Ag-CF_x_ composite film with φ = 0.15.

Optical characterization of IBS-deposited Ag-CF_x_ showed a plasmon resonance peak, falling between 424 ± 2 nm and 438 ± 2 nm, as shown in Fig. S1. AFM topography images were also acquired at samples deposited on glass slides (Fig. S2), showing a fine, granular, and in-plane homogeneous surface. Contact angle investigations of Ag-CF_x_ films exposed to oil and water droplets are reported in Fig. 2. Ag-CF_x_-treated textiles exhibited, at all φ values, an oil contact angle of about 100°, while analogous leather specimen displayed a water contact angle of more than 125°.

#### XPS surface chemical composition

**Figure 3:**
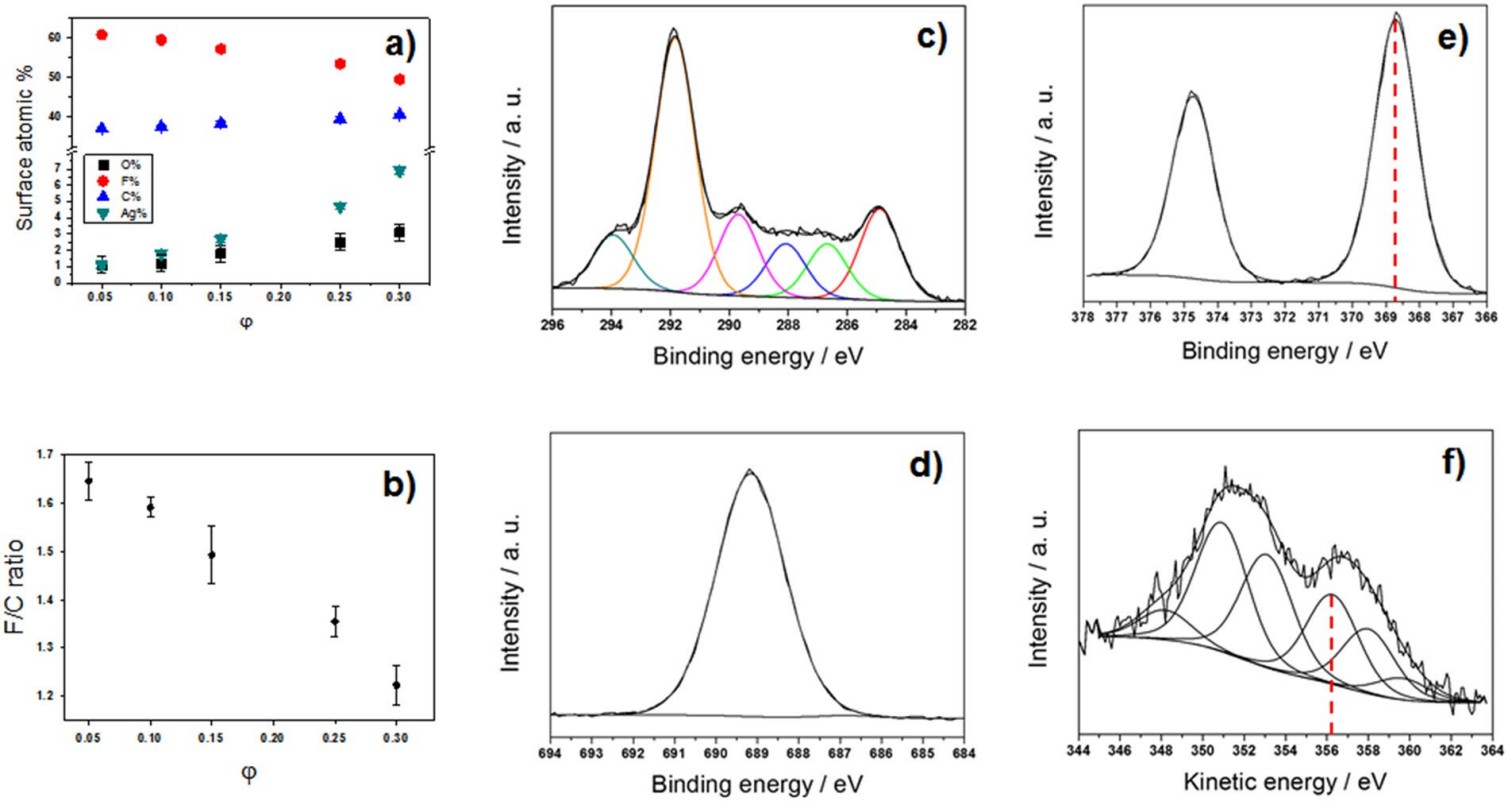
Surface atomic composition of Ag-CF_x_ nanomaterials as a function of the silver volume fraction φ (**a**). F/C ratio as a function of φ (**b**). Typical XP high-resolution regions for Ag-CF_x_ composites with φ = 0.25: C1s (**c**), F1s (**d**), Ag3d (**e**), AgM_4, 5_N_45_N_45_ (**f**).

Surface analysis of the materials was carried out by XPS. Elemental percentages are sketched in Fig. 3a, as a function of φ. As expected for this class of metal-CF_x_ films^14,30,49^, Ag surface atomic percentage increased upon increasing the inorganic loading. Additionally, at higher φ values, F% decreased significantly, while the C% followed an opposite trend. In Fig. 3b, the F/C atomic ratio is reported as a function of φ. Typical high-resolution XP spectra for a silver loading of φ = 0.25 are reported in Fig. 3c–f. Calculation of the modified Auger parameter^50^ (α′) was used to assess Ag chemical speciation. The two peak positions exploited for calculating α′ are highlighted in Fig. 3e–f by vertical dotted lines. In this study, modified Auger parameter resulted to 725.0 ± 0.2 eV, a value that is intermediate between the one expected for elemental Ag, and the one reported in literature for Ag_2_O^51^. This is reasonably due to a partial surface oxidation of the silver clusters when the composite film is exposed to air. The absence of AgF was confirmed, since the α′ value for this compound should have been equal to 722.8 ± 0.2 eV^52^.

### Study of silver ion release in physiological solution

**Figure 4:**
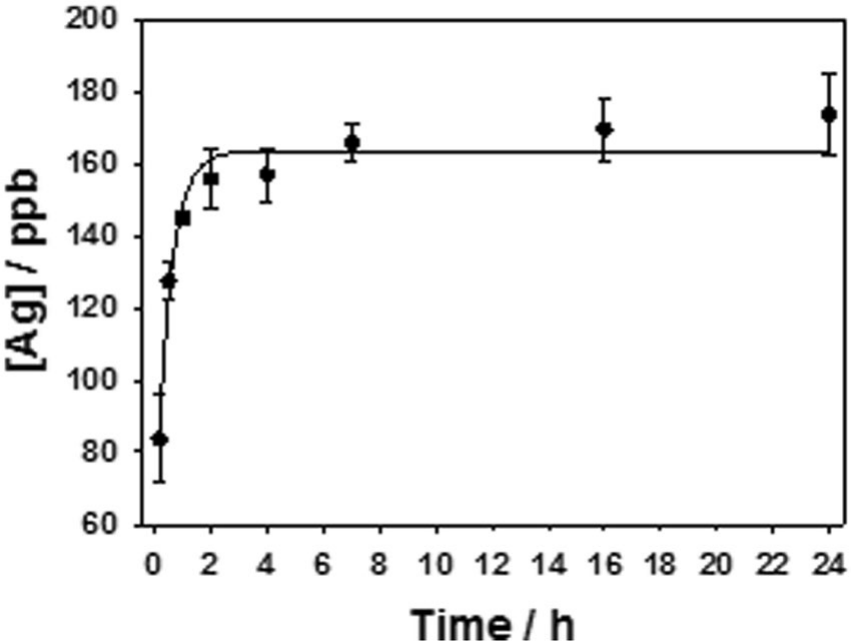
Silver ion release for an Ag-CF_x_ composite film with φ = 0.25 measured by ETAAS. Solid line shows data interpolation by means of a first order kinetics. R^2^ value resulted equal to 0.9806.

The investigation of metal-releasing properties was carried out evaluating the silver release kinetics from a model φ = 0.25 Ag-CF_x_ nanocomposite, in physiological solution by means of ETAAS. Figure 4 shows that the concentration of Ag^+^ species released into the physiological solution increased with time, reaching a plateau (160 ± 15 ppb) in about 2 h. XPS data on thin films after contact are reported in Table S1.

### Biofilm inhibition at coated ZnSe crystal

**Figure 5:**
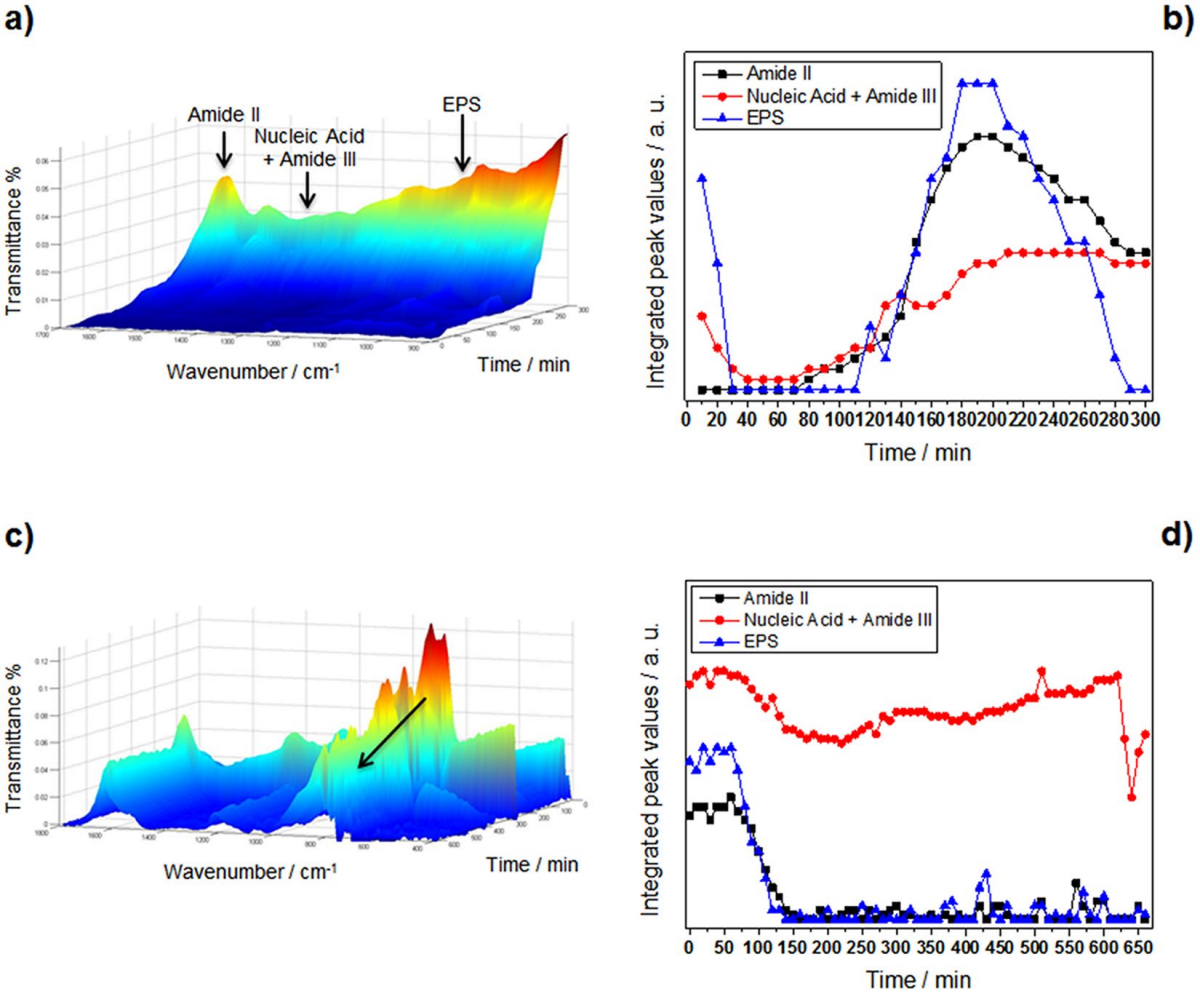
Temporal evolution of relevant IR bands for biofilm formation. (**a**) Control IR-ATR spectra of a *P. fluorescens* biofilm (arrows mark relevant IR bands) and (**b**) related integrated peak values (IPVs) as a function of time. (**c**) IR-ATR spectra of *P. fluorescens* biofilm on Ag-CF_x_-modified crystal (please note reversed time scale for better illustration; the arrow indicates the decrease in IR bands associated to EPS); (**d**) related IPVs as a function of time.

A suspension containing bacteria at the end of the exponential growth phase in sterile Luria-Bertani (LB) medium was used to initiate biofilm formation at the bare ZnSe crystal surface. During this step, accumulation of biomass at the ATR crystal surface and an increase of bacterial coverage is evident through the associated increase of the related IR absorption features. Changes of the band areas of amide II (1588–1483 cm^−1^), nucleic acid with amide III (1280–1196 cm^−1^), and extracellular polymeric substances (EPS) (1187–952 cm^−1^) are distinct indicators of bacterial attachment and associated biofilm growth^53^ (clearly visible in the control Fig. 5a). In Fig. 5b, the time dependence of areas of the relevant bands is plotted and, in fact, all bands monitored started growing after 120′ from the beginning of the experiment. The same experiment was repeated using the nanocomposite-film-modified ATR crystal (see Fig S3 and relevant text for details about the design and use of a mask to selectively deposit Ag-CF_x_ films at IR-inactive crystal regions). Spectra were recorded at 10-minute intervals at continuous flow conditions (flow rate 0.7 mL/min) for periods up to 11 h. Spectra obtained as a function of time are reported in Fig. 5c. A comparison between IR spectra at t = 0 and t = 11 h is shown in Fig. S4, with the corresponding band attribution summarized in Table S2. The intensity of all bands present in the spectra decreased with time (please note the reversed time scale in Fig. 5c for presentation). Plotting integrated peak values (IPVs) for the three spectral regions of interest vs. time (see Fig. 5d), it was observed that bands related to amide II and EPS reached almost zero within about 2 h, while the band associated to nucleic acid and amide III species was almost constant.

### AFM imaging of bacteria incubated at Ag-CF_x_ thin films

**Figure 6:**
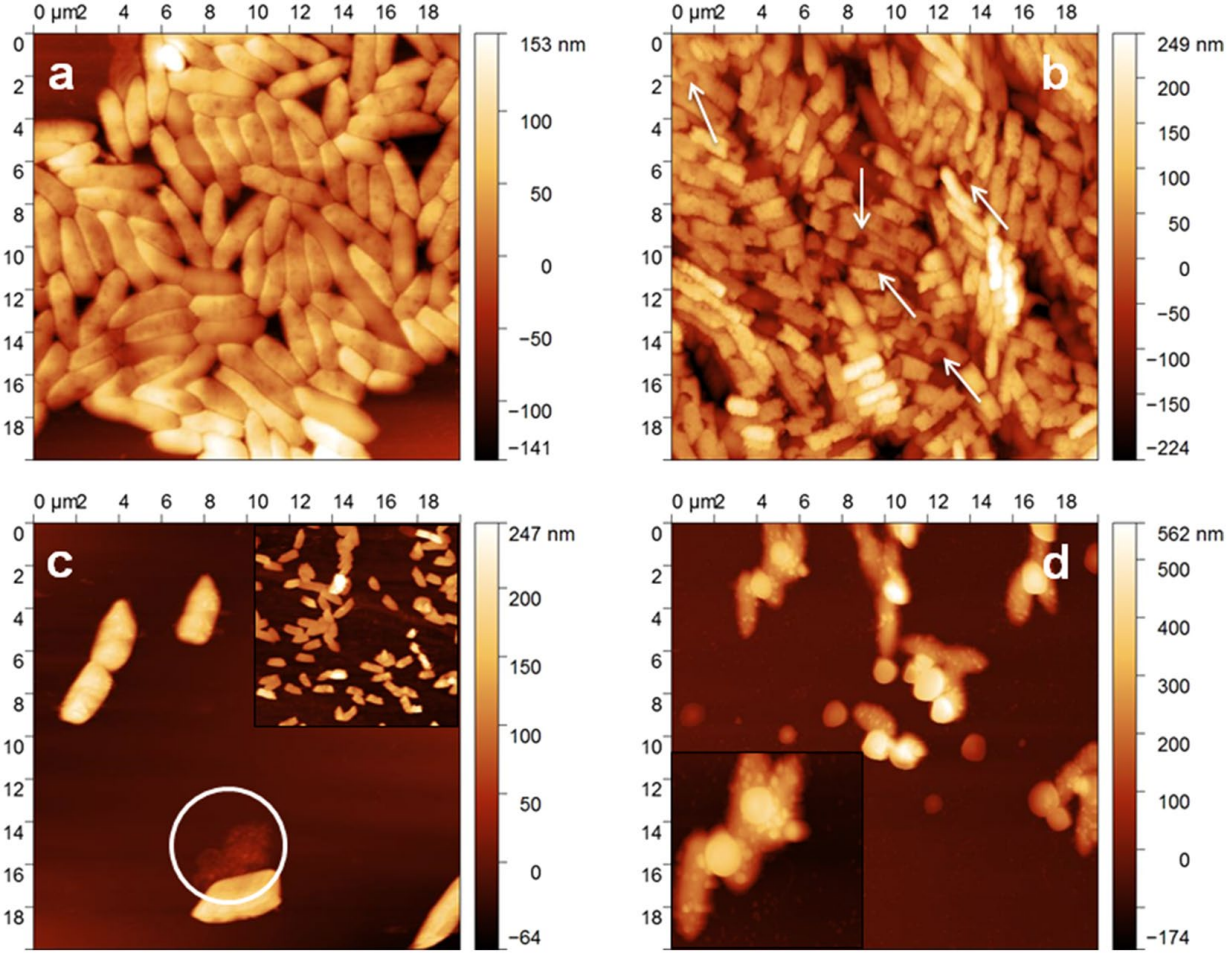
AFM topographies of *P. fluorescens* incubated on Ag-CF_x_ composite film, with φ = 0.25, as a function of the incubation time: t = 0 (**a**); t = 1 h (**b**); t = 4 h (**c**); t = 18 h (**d**). Arrows in panel **b** indicate membrane pits/craters.

The same bacterial culture used for IR-ATR experiments was also subjected to AFM studies. A washed bacterial pellet (see “Method” section for details) was deposited onto a silicon wafer and investigated by AFM. Figure 6a shows a micrograph obtained on living untreated bacteria. Figure 6b–d represent the AFM images of bacteria seeded on a φ = 0.25 Ag-CF_x_ thin film after a contact time of 1, 4, 18 h, respectively. Root mean squared roughness (RMS) data on the bacterial outer membrane surface are reported in Table 1, along with % increments. *P*. *fluorescens* RMS values were almost tripled after 4 h of incubation with respect to the values reported for untreated bacteria (t = 0). AFM on control sample showed healthy cells, with higher density.

**Table 1:** Obtained RMS roughness values of bacterial surface as a function of the incubation time.

Incubation time (h)	RMS (nm)	RMS % increment
0	22.4 ± 1.6	—
1	33.0 ± 2.3	+47%
4	62.1 ± 0.9	+177%
18	68.8 ± 0.8	+207%
Control (4 h)	28.9 ± 1.4	+29%

Data represent average values of 50 evaluated cells each. Control experiment was performed incubating bacteria on a CF_x_ thin film for 4 h.

**Figure 7:**
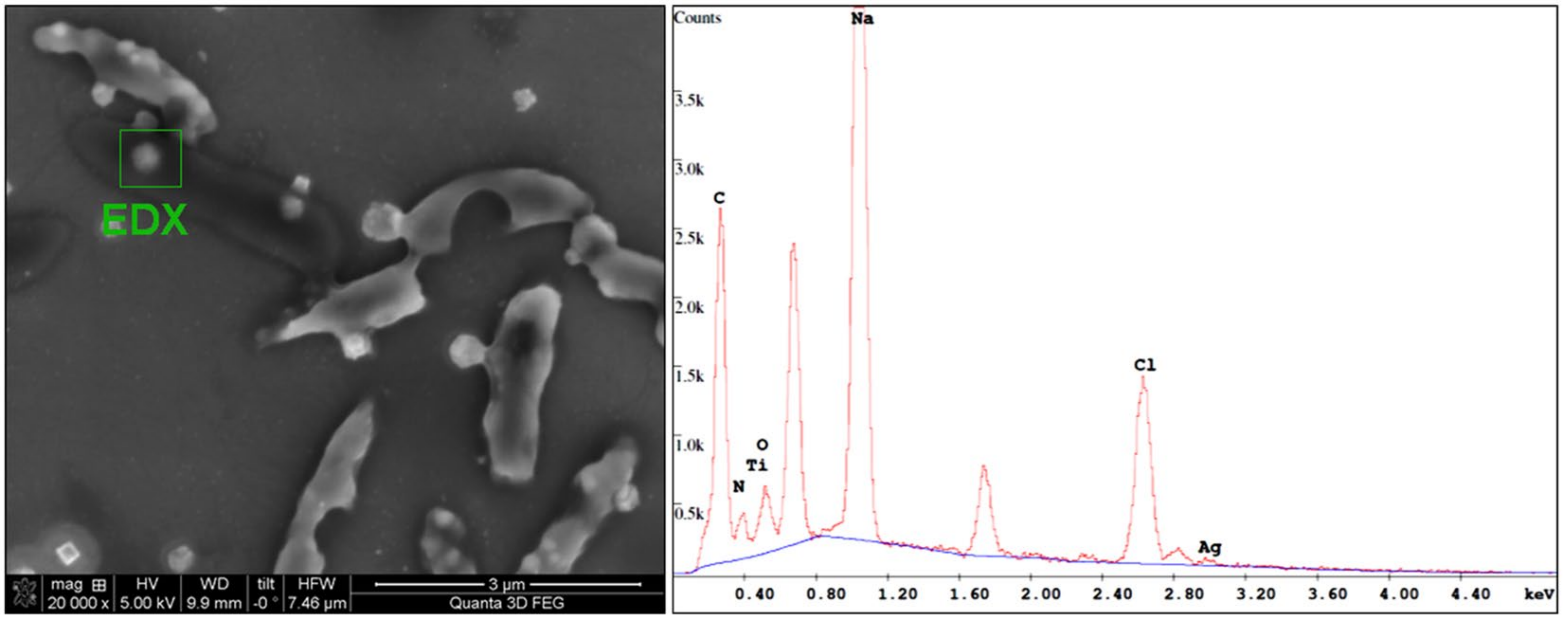
SEM micrograph on bacterial sample incubated on Ag-CF_x_ thin film for 18 h, and EDX spectrum of the highlighted area.

Scanning Electron Microscopy, coupled with energy dispersive X-ray (SEM-EDX) analysis (see Fig. 7) was applied to elucidate the nature of small, nanosized features apparent on the *P. fluorescens* biofilm sample of Fig. 6d, after an 18 h contact time. Corresponding SEM-EDX measurement on cluster-free areas of the same bacterial membranes is reported in Fig. S5 for comparison.

### Viability assays

**Figure 8:**
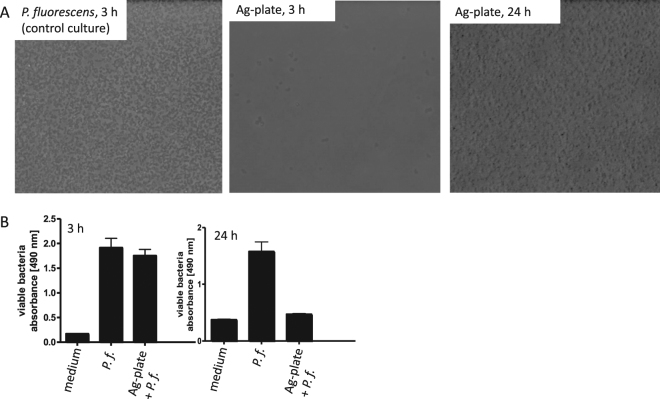
Viability of *P. fluorescens* after incubation with plates coated with Ag-CF_x_ composite film. (**A**) Control plate (left), Ag-CF_x_ composite film (middle) and after 24 h (right) analyzed by phase contrast microscopy. (**B**) Bar diagram for the viability test of the bacteria measured after 3 and 24 h of incubation with the Cell Titer 96^®^ aqueous non-radioactive cell proliferation assay (MTS assay). Values are given as mean ± S.D. (n = 3).

To test the antimicrobial effect of the plates coated with Ag-CF_x_ composite film, they were incubated with a suspension of *P. fluorescens* and the attachment of bacteria to the samples as well as the viability of the bacteria, which were grown in the presence of the samples, were analysed after 3 h and 24 h of incubation. As shown in Fig. 8A, only a very small number of bacteria were attached to the plates after 3 h while more bacterial material was attached after 24 h. However, analysis of the viability of the bacteria revealed that most of the bacteria growing with the plates were viable after 3 h, but not after 24 h of incubation (Fig. 8B). This result confirms the antibacterial effect of the Ag-CF_x_ composite film, which was observed by alternative methods before, and suggests that the bacterial mass, which might be associated with the plates after 24 h, might mainly consist of killed bacteria.

## Discussion and Conclusions

Ion beam co-sputtering is a very versatile and reproducible technique, enabling the reproducible deposition of films with a thickness ranging from few nm to few microns. Compared to evaporation techniques, IBS requires much lower operation temperatures. Unlike other sputtering approaches, such as magnetron and RF, IBS co-deposition from two independent targets, it provides an additional degree of freedom for the independent control of energy and current on each sputtering beam, tuning deposition rates for each constituent. IBS was here applied to the controlled deposition of Ag-CF_x_ nanoantimicrobials. Ion beam properties (energy and current), and hence the composite growth rate, could be easily chosen to obtain Ag-CF_x_ composites with a defined inorganic phase volume fraction (φ), as reported in Table S3. φ was calculated according to equation S1 (see SI). Transmission electron microscopy data in Fig. 1 showed that the composite is compact and homogeneous over a wide spatial range, with a regular in-plane distribution of almost monodisperse inorganic clusters, having an average size of about 9 nm and a relative standard deviation lower than 5%. Similar composites prepared by other physical technique showed larger cluster size (20–60 nm) at comparable metal loadings, generally reaching percolation limit at lower metal loadings^54^. Differently from similar materials, such as ZnO-CF_x_ composites, in which both cluster size and their surface density increased with ZnO loading^10^, no appreciable increase in cluster size was observed varying φ in the Ag-CF_x_ composites presented here. Some AgNPs showed resolved lattice spacing (see inset in Fig. 1c); selected area electron diffraction pattern showed the presence of distinct diffraction spots (Fig. 1d), attributable to both Ag^0^ and Ag_2_O. These evidences were interpreted as a partial NP surface oxidation induced by air exposure, leading to nanocrystalline, partially oxidised AgNPs. Similarly to AgNP size, which was almost independent on φ, also nanocomposites’ optical properties did not change significantly with the metal volume fraction (Fig. S1). This is a markedly different feature of IBS-deposited Ag-CF_x_ films as compared to analogous materials obtained by other methods, which showed a significant absorption shift with increasing φ^55^. AFM topography images shown in Fig. S2 revealed an in-plane homogeneous surface, with a slight increase in surface roughness upon φ. Contact angle investigations (Fig. 2) revealed that fluoropolymer anti-stain and water-repellent features are retained even when Ag-CF_x_ is deposited on textiles and leathers. XPS analyses of Ag-CF_x_ films showed a reasonable and marked dependence of the surface atomic percentages, as well as of the F/C ratio, on φ. Ag% and C% increased with this parameter, while F% and F/C ratio decreased. It is usually observed that the F/C ratio for IBS-deposited fluoropolymers is significantly below 2 (value expected for a standard Teflon^®^ surface). This is related to Teflon-like moieties rearrangement into a variously branched organic layer. Co-deposition of metal nanophases increases the CF_x_ polymer cross-linking degree, as well^56^. In this study, the extent of polymer defluorination depended almost linearly from φ. Interpretation of high resolution XP spectra was made by standard curve-fitting protocols^14^. Six photoelectron components were used to resolve the C1s signal (Fig. 3c). Details on peak positions and attributions are reported in Table S4 and related text. Noteworthy, F1s high-resolution spectrum presented a single component, located at 688.8 ± 0.3 eV, which was identified as organic fluorine (C-F)^30^. Unlike other composites made of inorganic clusters embedded in a Teflon-like matrix^10,14^, here the F1s signal component ascribable to inorganic fluorides (expected at about 684.8 ± 0.2 eV^57^) is completely absent at all metal loadings. It is very important to point out that the absence of toxic/irritant inorganic fluorides^58^ makes the application of these composites in real-life products more straightforward, ruling out *a priori* toxicological issues related to AgF^2^. Ag chemical speciation was addressed by studying AgM_4,5_N_45_N_45_ Auger (Fig. 3f) and Ag3d photoelectron signals (Fig. 3e), as outlined in the results section. It is very important to note that the surface analysis data allowed excluding the presence of potentially toxic silver fluorides, the Ag NP surface being composed of Ag_2_O and Ag species.

ETAAS was used to assess the Ag-CF_x_ ion-releasing properties. Ag^+^ release kinetics were measured on a model φ = 0.25 Ag-CF_x_ nanocomposite (Fig. 4). As expected for this class of nanoantimicrobials, the concentration of Ag^+^ species released into the physiological solution increased with time and reached a plateau in about 2 h. XPS data on thin film after ETAAS experiment confirmed these findings, as outlined in Table S1. Experimental data were well interpolated by a 1^st^ order kinetic curve, having a rate constant equal to 1.9 ± 0.5 h^−1^. It is noteworthy that silver ion concentration at t = 0 was not equal to 0. This is in agreement with the presence of partially oxidized silver species on the NP surface, which are readily soluble when the composite is let in contact with the aqueous matrix. Moreover, no significant difference was observed in plateau values with varying AgNP inorganic loading into composites. This is reasonable since [Ag^+^] plateau concentration was close to the solubility limit of AgCl in the contact solution.

ATR-IR was used to study biofilm formation/inhibition on the surface of both bare and Ag-CF_x_-modified ZnSe crystals. Bands associated to amide II (1588–1483 cm^−1^), nucleic acid with amide III (1280–1196 cm^−1^), and extracellular polymeric substances (1187–952 cm^−1^) were chosen as distinct indicators of bacterial attachment and associated biofilm growth^53^. During initial biofilm growth stages, accumulation of biomass at the bare ATR crystal surface and an increase of bacterial coverage is evident through the associated increase of the related IR absorption features (Fig. 5a,b). It is immediately observable that EPS and amide II levels significantly increased as a function of time. The level of nucleic acid/amide III band remained almost constant after 2 h. These findings suggest that less nucleic acids were synthesized compared to polysaccharides during the initial attachment of bacteria. This is consistent with findings of Humbert *et al*.^44^, who observed that this evolution of amide band II is characteristic of bacterial colonization at the crystal surface. After approximately 4 h, the amount of EPS and amide II appeared reduced, increasing the surface coverage, and therefore the unoccupied area of the waveguide surface could be considered negligible. The same experiment was then repeated on the Ag-CF_x_-modified ATR crystal (Fig. 5c–d). In this case, the nanoantimicrobial coating was carefully deposited only on IR-inactive regions of the waveguide (see Fig. S3c). The sampled biofilm spots were therefore exposed only to Ag^+^ species released by neighbouring regions, without being in direct contact with antimicrobial NPs. This approach allowed for simplifying the system, reducing the number of possible bioactivity mechanisms only to the biofilm exposure towards metal ions. Under these conditions, it was observed that bands related to amide II and EPS reached almost zero within about 2 h. This indicated complete biofilm eradication from the waveguide surface. On the contrary, the intensity of the band associated with nucleic acids (1280–1196 cm^−1^) remained almost constant within the experimental time frame. It is well known^59,60^ that a strong antimicrobial effect is generally associated to cell death and lysis. The high abundance of this intercellular component is therefore directly associated with an almost complete disruption of *P. fluorescens* bacterial membranes.

The same bacterial culture used for IR-ATR experiments was subjected to AFM studies. Untreated bacteria appeared well-packed and ordered, not stacked, with defined contours, and with a low surface roughness (Table 1). On the contrary, bacteria seeded on a φ = 0.25 Ag-CF_x_ film exhibited a substantial stacking after a contact time of 1 h. Indeed, bacterial stacking is indicative for elevated stress conditions^61,62^. Moreover, a significant change in cell morphology of *P. fluorescens* could be observed after incubation on the silver-fluoropolymer nanocomposite (see arrows in Fig. 6b). AFM images clearly revealed several deep craters at the bacterial surface resulting from membrane disruption. The latter appeared rougher (for RMS values, see Table 1) compared to bacteria seeded on bare CF_x_, even though typical rod-shaped morphology was almost retained. Pits in the cell membrane may be associated with a strong electrostatic interaction between silver ions released in the bacterial suspension and the *P. fluorescens* bacterial cell wall^61^. This is in agreement with the hypothesis that Ag-CF_x_ antimicrobial action results from Ag^+^ release in solution. Occurrence of pits/craters on bacteria, when they are in contact with (silver) nanoantimicrobials, is a well-known phenomenon, reported in literature for the first time more than 10 years ago^63^. After 4 h of incubation (Fig. 6c), mostly lysed cells were evident. Loss of cytoplasm was sometimes clearly noticeable, as highlighted in the image with a circle. Deep and irregular grooves at the cell surface were also present. Hence, with an increase in incubation time, groves and pits caused by ions led to the complete disruption of the cell wall and loss of intracellular matrix. This observation confirmed that Ag^+^ ions may indeed fragment bacterial membranes, thereby leading to a substantial volume reduction caused by the loss of cytoplasm, and finally, to cell death^64^. For the longest incubation time, the sample appeared profoundly different (Fig. 6d): after 18 h, *P. fluorescens* bacteria were uniformly covered by nanosized features, probably containing silver. This hypothesis was confirmed by SEM-EDX analyses of the same sample (Fig. 7). In fact, Ag traces were evident along with other elements like Na, C, Cl, O, and N, related to the biological sample. The presence of inorganic nanophases on the bacterial surface could be attributed either to partial release of entire AgNPs from the thin film or to precipitation of insoluble Ag salts onto the bacterial surface. Experiments aimed at outlining the possible release of entire AgNPs were therefore carried out, as outlined in the experimental section. No AgNPs were visible at any magnification; on the contrary, submicron features ascribable to electrolyte crystals were evident (Fig. S6). Therefore, the clusters visible in SEM pictures of bacterial surfaces were attributed to local precipitation of insoluble silver species, such as AgCl. Absence of Ag on uncovered membrane regions was confirmed by EDX, as well (Fig. S5). Moreover, it is important to note that the absence of nanoparticle release -as confirmed herein- rules out the nano-toxicological risk associated with free AgNPs dispersed into aqueous contact fluids. NPs extraction induced by contact with bacteria cannot be completely excluded; however it is highly unlikely considering that metal grains are known to penetrate the “soft” polymer matrix^65^ and are less abundant on the outer surface layers. Moreover, if lipophilic interactions are claimed, the Teflon-like material should attract NPs much better than the bacterial membrane; if hydrophilic interactions are considered, water should be more efficient than bacteria in this extraction.

In conclusion, Ag-CF_x_ nanocomposites with different inorganic fractions were here deposited by IBS using well controllable deposition parameters. TEM and XPS characterizations showed differences with previously reported metal/metal oxide-fluoropolymer nanocomposites. In particular, Ag cluster size was below 10 nm without aggregation or percolation paths at high loadings, which is distinctly different from the behavior of similar metal-CF_x_ materials. The presence of toxic metal fluorides was excluded, thus supporting real-life applications. ETAAS quantification of ionic release in physiological solution was also performed, showing that Ag^+^ concentration was sufficiently high to provide antibacterial efficacy and significantly lower than the limit of ~1 ppm^66^, which is generally considered a threshold value for Ag^+^ toxicity towards humans. Bioactivity was assessed against *P. fluorescens* microorganism: its growth was monitored in molecular detail via flow-through IR-ATR experiments, whereby characteristic IR bands indicative of biofilm development were clearly identified and acquired as a function of time. Subsequently, biofilm inhibition was studied using Ag-CF_x_-modified ATR waveguides. The nanocoating was deposited at the ZnSe waveguide surface after identification of its IR-inactive areas. This procedure prevents that the IR signals are convoluted with absorption features of the polymer film. Moreover, this allowed decoupling antimicrobial effects generated by Ag^+^ from cellular stress induced by a direct contact of AgNPs and bacteria. These results were corroborated by AFM studies. The analytical approach herein presented for the study of nanomaterial-biofilm interactions will prospectively be of great importance for understanding the different nanoantimicrobials’ bioactivity mechanisms. This approach could allow in the next future to decouple ion-mediated effects from nanoparticle-mediated and direct contact ones, finally shading light on a controversial matter^2^.

## Methods

### Ion beam sputtering deposition of Ag-Teflon thin films

Silver–fluoropolymer (Ag-CF_x_) nanocomposites were deposited on different substrates by co-sputtering a PTFE target (GoodFellow LTD) and a pure Ag target (Gambetti-CERAC, 99.999%) with Ar^+^ ion beams, at room temperature and at a pressure of 10^−2^ Pa. A “customized” dual-ion-beam system, already described elsewhere^10^, was used to deposit the films. The volume fraction (φ) of the inorganic phase into the organic film was ranged between φ = 0.05 and φ = 0.30. For further details on φ calculations, please refer to SI (Eq. S1). Films thickness was 150 nm, unless otherwise stated.

### TEM and XPS characterizations

30-nm-thick films for TEM characterization were directly deposited onto carbon-coated Cu grids (300 mesh, Agar Scientific). All samples were observed at 120 kV, with a FEI Tecnai T12 TEM. Selected area electron diffraction (SAED) measurements were performed using the same conditions, in dark field. Ag nanoparticle size analysis and SAED pattern studies were performed with ImageJ software (http://imagej.nih.gov/ij/).

TEM analyses aimed at outlining the possible release of entire nanoparticles were based on the following protocol. A glass slide modified by an Ag-CF_x_ thin film (φ = 0.25) was exposed to an aqueous solution resembling bacteria culture medium, deprived of organic components. After 18 h of contact, the solution was sampled and deposited onto Formvar^®^/carbon-coated Cu grids (300 mesh, Agar Scientific) for TEM measurements.

XPS studies were performed on films deposited on 3 × 2 cm silicon slides (SiMat Silicon Materials, <110>, 300 μm, undoped, single-side polished) using a Theta Probe spectrometer from Thermo Fisher Scientific. XPS measurements were performed at least in triplicate using a monochromatic 300 μm X-ray source. The acquisition time of the whole spectral set was adjusted as the best compromise between signal-to-noise ratio and minimal exposure time to radiation. Sample damaging was excluded by comparing spectra of the same transition, which were acquired at the beginning and at the end of each analysis. A flood gun was used to minimize surface charging, using an Ar pressure of ~10^−8^ mbar. Detailed spectra analysis was performed by the commercial Thermo Avantage^©^ software (v. 5.937, 2014) from Thermo Scientific. Surface composition was determined by considering integrated peak areas (after Shirley background removal) and Scofield sensitivity factors. The same peak lineshape parameters (Gaussian/Lorentzian ratio and full width at half maximum) values were employed for the curve fitting of components belonging to the same high-resolution spectrum. Spectra were corrected for charge compensation effects by offsetting the binding energy relative to the –CF_2_– component of the C1s spectrum to 291.8 eV.

### ETAAS measurements

The determination of the amount of silver released from the nanocomposites into an aqueous contact solution was carried out through atomic absorption analyses. Silver release experiments were performed putting coated glass slides in contact with 500 μL of phosphate buffer saline solution (pH 6.8, I = 0.1, Sigma Aldrich, Trace-SELECT^®^, for trace analysis, ≥99.999%, anhydrous) for defined sampling times up to 24 h. 15-μL aliquots of the contact solutions were sampled and analysed via ETAAS after dilution with 0.2% HNO_3_ (Sigma Aldrich, 65–71%, Trace-SELECT^®^ Ultra, for ultratrace analysis). A Perkin-Elmer PinnAAcle AS 900Z double beam spectrometer, equipped with a silver hollow cathode lamp, a longitudinal Zeeman system, and a transversely heated graphite tube was used for ETAAS analysis. A calibration curve was established by dilutions of a silver standard for AAS (Perkin Elmer, Ag pure single-element standard, 1000 µg/mL in 2% HNO_3_). All measurements were carried out in triplicate. See SI for details on data analysis and calculations of ion release kinetics.

### Bacterial strain and culture conditions

The non-pathogenic strain *P. fluorescens* ATCC 13525 was obtained from the Institute of Microbiology and Biotechnology, Ulm University (Germany). Cells at the end of the exponential growth phase (refer to SI for further details) were harvested and resuspended in 1:49 diluted LB medium (0.5 g/L). For AFM measurements, the bacterial pellet obtained by centrifugation was washed twice with sterile Milli-Q, and re-suspended in sterile Millipore water. Then, this aqueous microbial suspension was pipetted onto Ag-CF_x_ - coated glass slides and let in contact for defined times (1 h, 4 h, and 18 h), then recovered and used for AFM sample preparation (vide infra). Experiments were performed at laboratory temperature (20 °C).

### Characterization of *P. fluorescens* biofilm by IR-ATR spectroscopy

For all experiments, a horizontal 45° six-reflection ZnSe crystal (Harrick Scientific, Pleasantville, NY) with dimensions of 72 × 10 × 6 mm was used as waveguide. The refractive index of ZnSe is 2.4 at 1000 cm^−1^, as reported by the manufacturer. The refractive index of the optically rare medium was estimated around 1.4 ± 0.1 based on the refractive index of bacterial components in the mid-infrared region in aqueous environment at 2850 cm^−1^ ^67^. Ozone treatment was used to clean the ZnSe crystal, exploiting intense ultraviolet light (wavelength: 185 nm and 254 nm), in the presence of O_2_. Localization of waveguide IR active areas was performed as reported in SI. The crystal was mounted via rubber gasket into a flow cell with an internal volume of 1.75 mL and a surface contact area of 5.2 cm^2^. The flow cell assembly was placed in the sample chamber of the infrared spectrometer (Bruker IFS 66/S, Bruker Optics, Ettlingen, Germany). A peristaltic pump (Watson Marlow Series 400, Cornwall, UK) was used to circulate liquids through the system, at a flow rate of 0.7 mL/min, which resulted in a residence time within the flow cell of ~150 s.

Prior to each measurement, the ATR flow cell was cleaned with 70% EtOH (VWR, 95–97%_v/v_, technical grade) for several hours, and afterwards rinsed with sterile water for 1 h. In order to establish a conditioning film at the crystal surface and to yield a background spectrum for the subsequent bacterial spectra, sterile LB medium (0.5 g/L) was introduced into the IR-ATR flow cell and flushed for 4 h. Afterwards, the LB medium was replaced by a bacterial suspension containing ~10^8^ CFU/mL in sterile LB medium (0.5 g/L) for 2 h. This period of time was found to be sufficient for initiating attachment of *P*. *fluorescens* bacteria at the ZnSe crystal surface. After 2 h of initial attachment of *P. fluorescens*, sterile LB medium (0.5 g/L) was pumped through the ATR cell for a period up to 20 h and the development of biofilms was monitored. No changes in IPVs were appreciable after 11 h. The resolution of the obtained IR spectra was 4 cm^−1^. 100 averaged spectra were recorded every 10 minutes. All spectra were registered at 21 ± 1 °C in an air-conditioned room. Water vapour subtraction and baseline correction were applied to the entire spectral data set. Data processing was accomplished via the Bruker OPUS™ 7.1 software. We indicated as “bare” the ZnSe crystal that was used as received from the producer, with no coatings/surface treatments. Analogously, we named “treated” the crystal modified with the antimicrobial Ag-CF_x_ thin film deposited by IBS. Experimental conditions here reported were used for experiments performed with both substrates.

### AFM imaging of bacteria incubated at Ag-CF_x_ thin films

500 μL of bacterial suspension, prepared as described before, were pipetted onto sterilized (i.e. autoclaved) Ag-CF_x_-coated glass slides (φ = 0.25) for defined times (1 h, 4 h and 18 h). After each incubation time, 2 μL were taken from the bacterial solution in contact with the Ag-CF_x_ film, deposited onto silicon substrates (TopSil, <100>, 240–260 μm, undoped, double-side polished), and dried for 2–3 h under a clean bench. Solution deposited on silicon slides for AFM measurements was sampled after a proper mixing step, performed by means of a pipette tip, thus randomizing the sampling step. In order to limit problems related to long-time storage, all samples were analysed within 10 h from their preparation. Each Si substrate was rinsed with acetone (Merck Millipore, for analysis), 2-propanol (VWR Chemicals, technical grade) and Milli-Q water before use, to remove organic contamination. Afterwards, they were sterilized via dry autoclaving procedures. Topography images were acquired using a Keysight AFM system Model 5500 (Keysight Technologies, AZ, USA). AFM images were obtained in dynamic mode, in air, using polygonal Si probes with a typical tip radius of 8 nm (Nanoworld NCL-W Pointprobe^®^). Further details are reported in SI. All AFM images were analysed using the freely available software Gwyddion 2.41 (http://gwyddion.net/).

### Viability assay

A suspension containing *P. fluorescens* at the end of the exponential growth phase in sterile LB medium was applied to plates coated with Ag-CF_x_ composite film in plastic bacterial culture dishes and the samples were incubated at 27 °C. For control, the bacteria were grown under the same conditions without plates coated with Ag-CF_x_ composite film. After 3 h and 24 h of incubation, the plates were taken out off the medium and analyzed by phase contrast microscopy for attached bacteria. Pictures of the samples were taken using a Zeiss Axiovert 40CFI microscope (Oberkochen, Germany) with a Jenoptik progress C10 CCD camera (Jena, Germany). In addition, a picture of the control culture was taken after 3 h of incubation. The viability of the bacteria was measured after 3 h and 24 h of incubation with the Cell Titer 96^®^ aqueous non-radioactive cell proliferation assay (MTS assay) from Promega (Mannheim, Germany), according to the manufacturer’s instructions. For this purpose, aliquots from the *P. fluorescens* culture growing in the absence (indicated as *P.f*.) or in the presence of the plates coated with Ag-CF_x_ composite film (indicated as Ag-plate + *P.f*.) were analyzed in the MTS assay and the absorbance was measured at 490 nm by using a plate reader and the values are given as arbitrary units. Sterile LB medium alone was taken for control. Values are given as mean ± S. D. (n = 3).

## Electronic supplementary material

Supplementary Information
