# Supplementary material for: Inhibiting P. fluorescens biofilms with fluoropolymer-embedded silver nanoparticles: an in-situ spectroscopic study
Source: Sci Rep. 2017 Sep 19;7:11870. doi: 10.1038/s41598-017-12088-x (PMC5605679; doi:10.1038/s41598-017-12088-x)
Supplement: Supplementary file 1 — Supplementary Information [file 41598_2017_12088_MOESM1_ESM.pdf]

Electronic Supplementary Information

Inhibiting P. fluorescens biofilms with fluoropolymer-embedded silver nanoparticles: an in-situ spectroscopic study

M.C. Sportelli, E. Tütüncü, R.A. Picca, M. Valentini, A. Valentini, C. Kranz, B. Mizaikoff, H. Barth, N. Cioffi

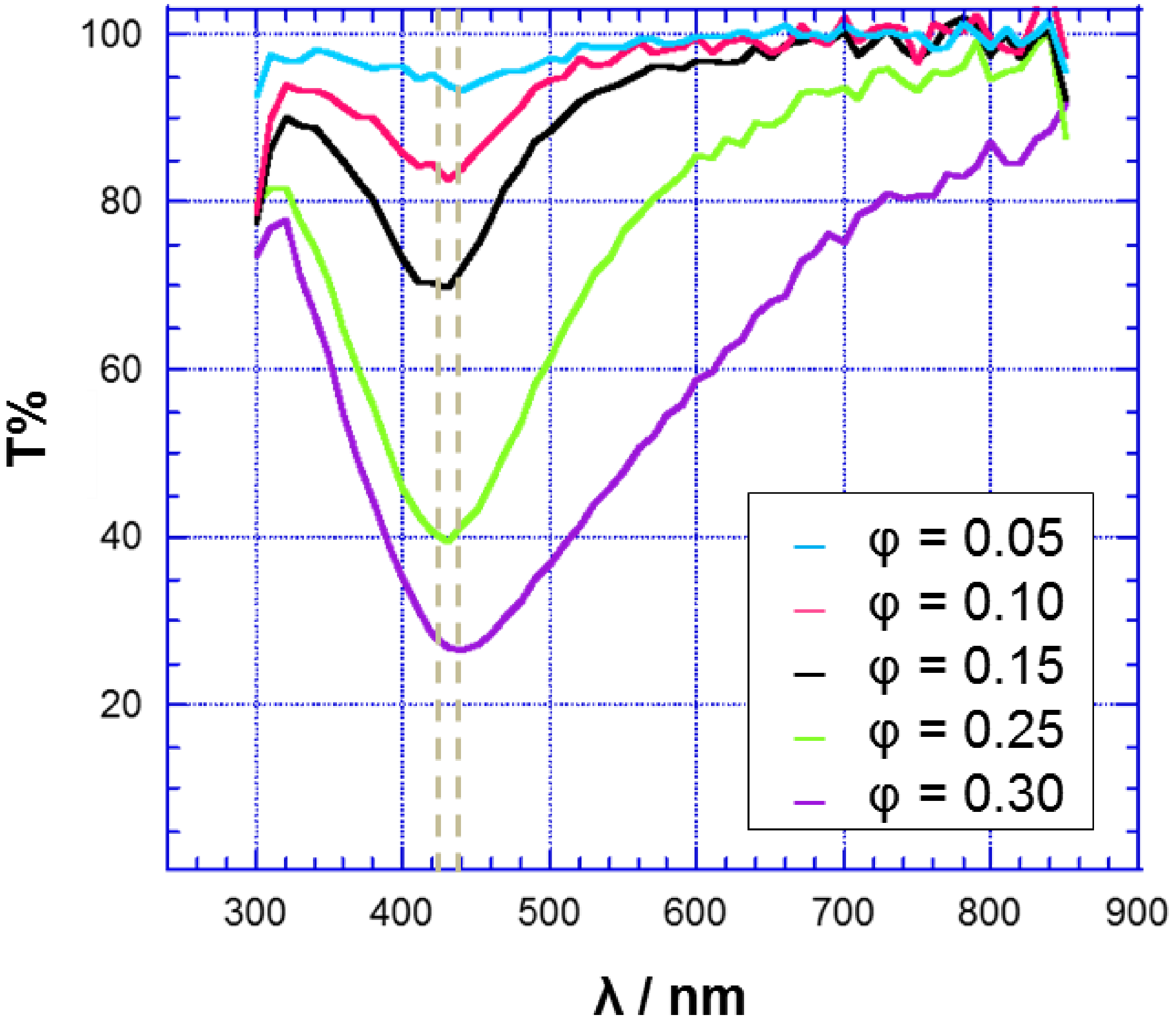

Fig. S1. Transmittance of Ag-CF_x thin films as a function of φ. Grey dotted lines outline plasmon resonance peak positions, ranging from 424 ± 2 to 438 ± 2 nm.

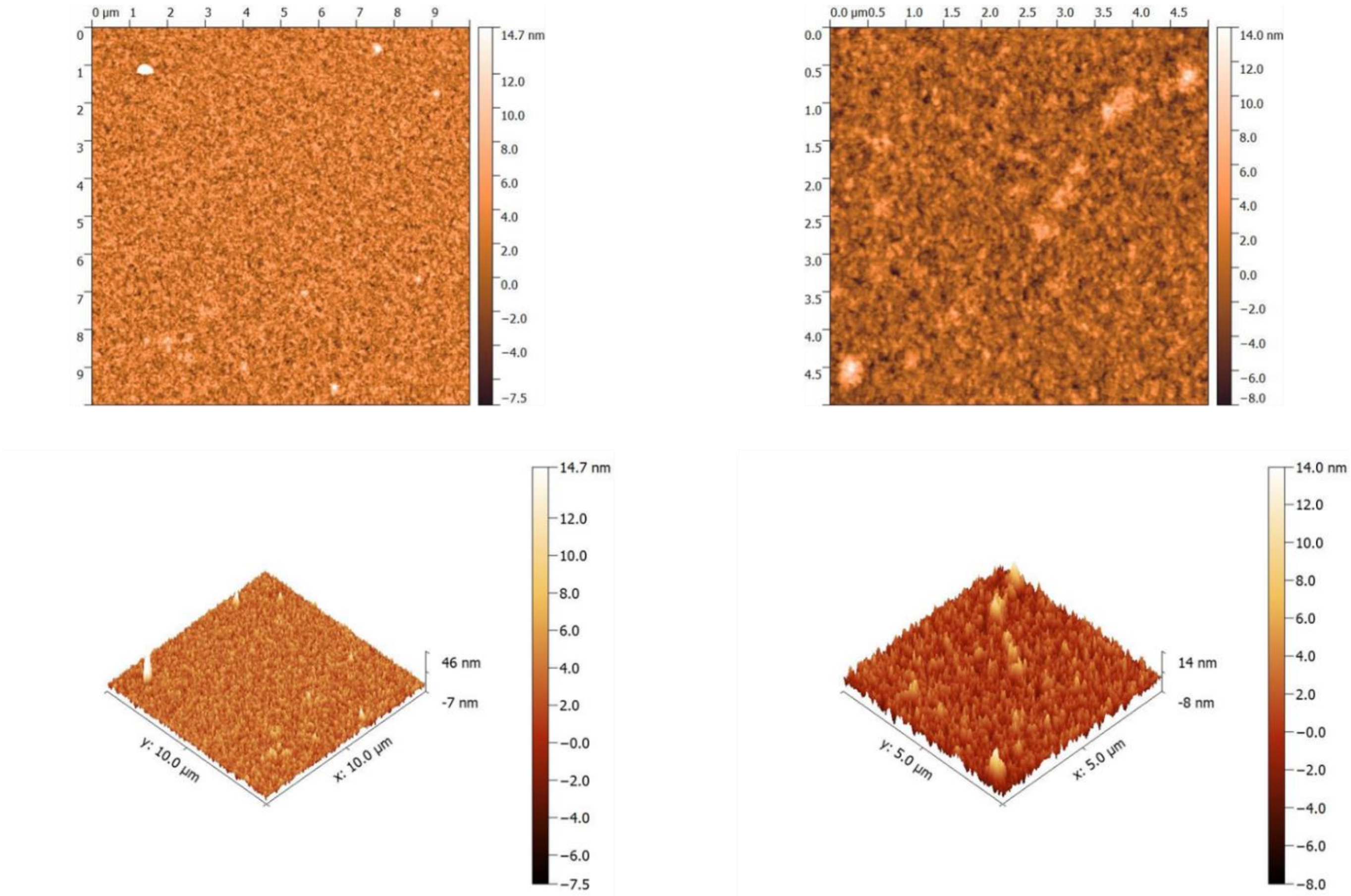

Fig. S2. AFM topography images of an Ag-CF_x composite film with φ =0.25 at two different magnifications (upper panel) and corresponding 3D views (lower panel) recorded in tapping mode.

Tab. S1. Surface elemental characterization of a bare Ag-CF_x composite film with φ =0.25, and after 2h and 24 h of immersion in **a** 0.5% NaCl solution.

	**t = 0**	**t = 2 h**	**t = 24 h**
**C**	40.0 ± 0.5	43.0 ± 0.5	41.5 ± 0.5
**F**	53.9 ± 0.5	52.3 ± 0.5	53.6 ± 0.5
**Ag**	3.6 ± 0.5	1.7 ± 0.5	1.8 ± 0.5
**O**	2.5 ± 0.5	3.0 ± 0.5	3.1 ± 0.5
**Cl**	-	< 0.5	< 0.5

Crystal coating: mask design for IBS deposition

The first step toward the development of an Ag-CFx-modified ZnSe crystal was to determine the hot spots of the individual internal reflections along the multi-reflection ATR crystal. For the determination of active IR regions along the waveguide, a procedure published by Dobbs et al.[1] was followed. IR-ATR spectra of drop-cast poly(styrene-co-butadiene) (PSCB, Aldrich, 45% styrene) at uniform distances were recorded. This way, the individual IR-active spots were visualized. Based on this 'IR activity map', a deposition mask was designed for IBS deposition of the φ = 0.25 Ag-CF_x thin film at the inactive areas of the ZnSe crystal (see Fig. S3a-b). Prior to the IBS deposition, the crystal surface was cleaned by prolonged sonication in 2-propanol and exposure to ozone plasma. The deposition process was carried out as reported before to obtain a φ = 0.25 Ag-CFx thin film with a thickness of 150 nm. The modified areas of the crystal were immediately visible to the naked eye, as shown in Fig. S3c. AFM topography of the coated regions of the ATR crystal (data not shown) revealed that the thin film morphology was the same obtained on glass slides (RMS values were 1.43 ± 0.07 nm and 1.48 ± 0.07 nm, respectively). The obtained waveguide was then mounted into the flow-through cell and used for subsequent experiments.

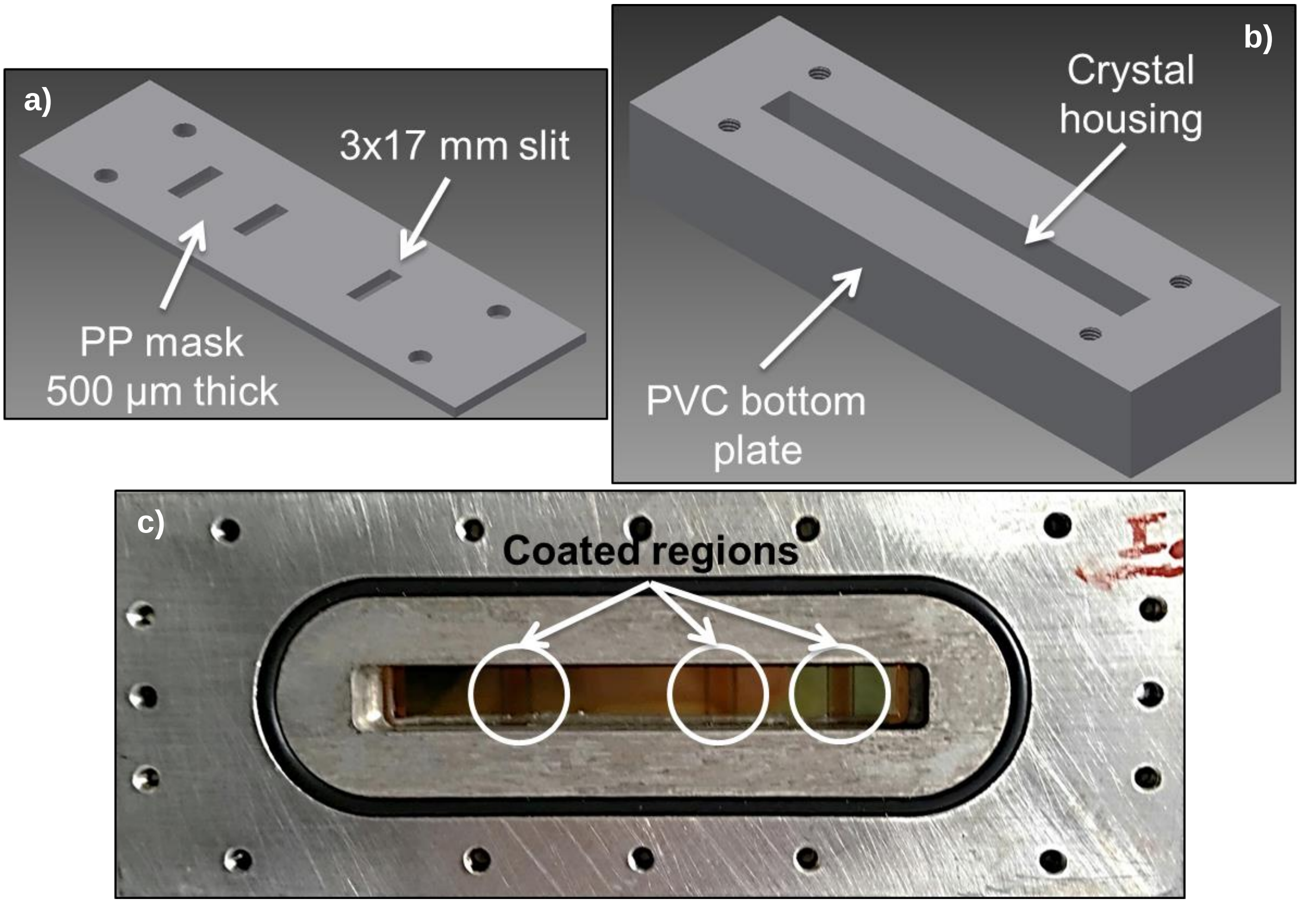

Fig. S3. Top mask (a) and crystal housing (b) for IBS deposition of Ag-CFx thin film on the waveguide surface. Modified ZnSe crystal after IBS process (c).

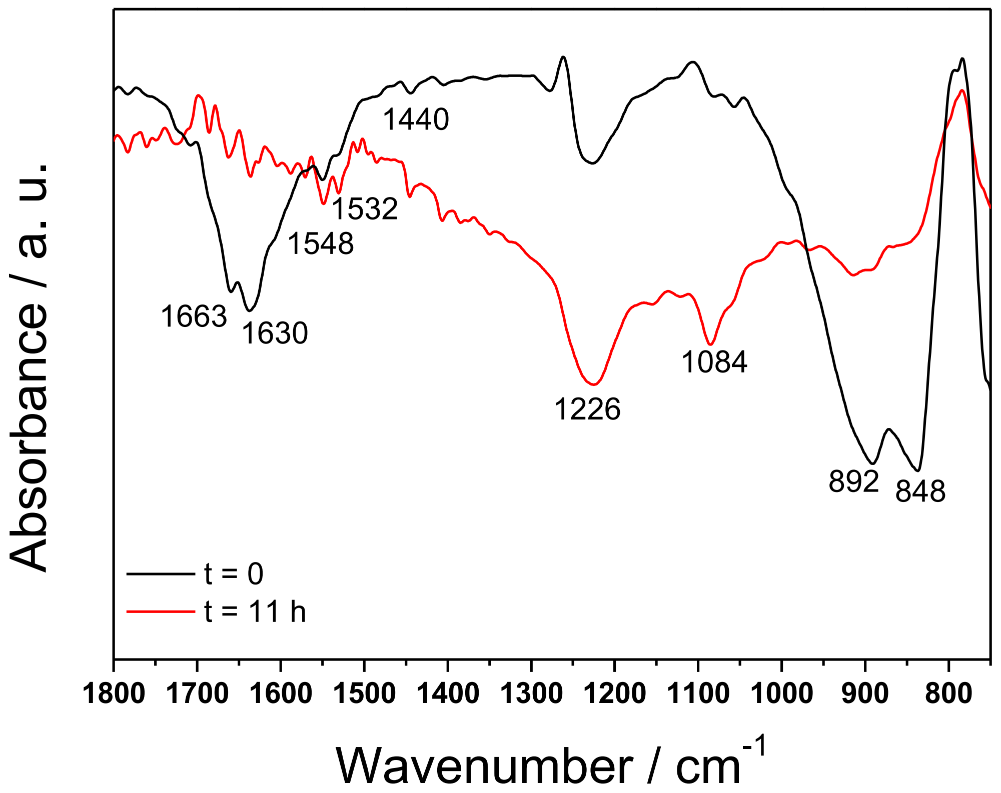

Fig. S4. Overlay of ATR-IR spectra at t = 0 and t = 11 h.

Tab. S2. Attributions of typical IR signals for P. fluorescens [2]. Error on peak positions is ± 2 cm^{-1}. ν indicates symmetric (s), or asymmetric (a) stretching; δ represents vibrational distortions.

**Signal Attribution**	**Position (cm^{-1})**
Amide I ($\nu_{C=O}$ coupled with δ_{N-H} and δ_{H2O})	1165-1630
Amide II (δ_{N-H} coupled with ν_{C-N})	1550-1525
δ_a ($_{CH2}$)	1440
Amide III (ν_{C-N} coupled with δ_{N-H})	1320-1280
Nucleic acids ($\delta_{a\ (PO2-)}$, $\delta_{s\ (PO2-)}$)	1226, 1084
δ_a (C-C; C-H) aromatics	890-850

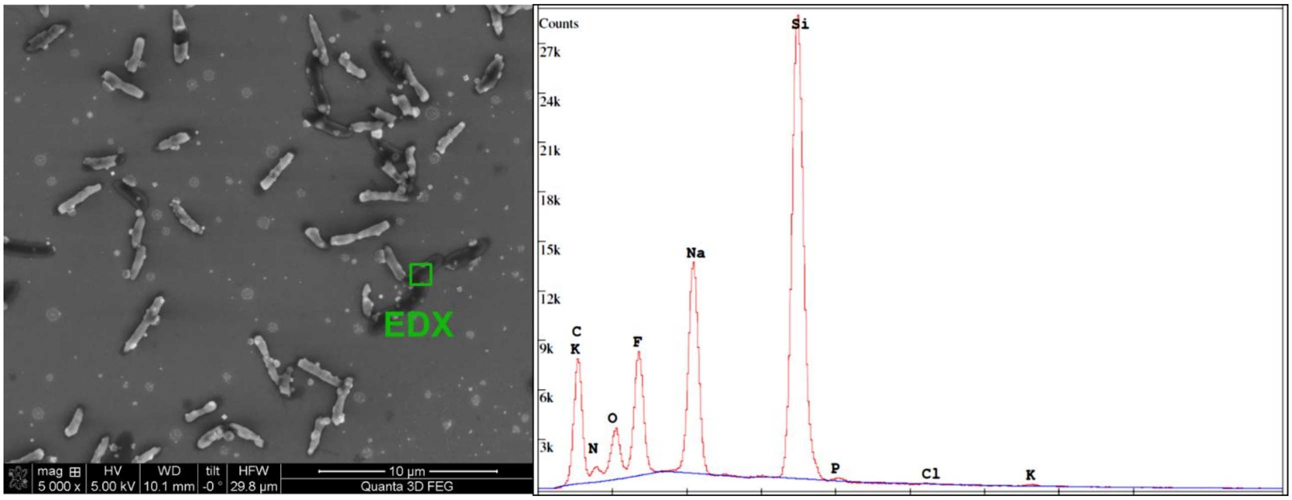

Figure S5. SEM micrograph on bacterial sample incubated on Ag-CF_x thin film for 18 h, and EDX spectrum of the highlighted area, where no inorganic clusters are visible.

Tab. S3. IBS experimental parameters relevant to Ag-CF_x nanocomposites as a function of the metal volume fraction.

φ	IB CFX (mA)	VB CFX (V)	IA Ag (mA)	VA Ag (V)
0.05	40	1000	20	350
0.10	40	1000	25	400
0.15	40	1000	30	500
0.25	40	1000	35	600
0.30	40	1000	35	700

Eq. S1: Detail of φ calculation. V_{PTFE} and V_{Ag} are the deposited volumes of PTFE and Ag, and r_{PTFE} and r_{Ag} are the deposition rates of the two materials, experimentally measured for any deposition run and expressed as film thickness per second.

$$\varphi = \frac{V_{Ag}}{V_{PTFE}+V_{Ag}} = \frac{r_{Ag}}{r_{PTFE}+r_{Ag}} = \frac{r_{Ag}/r_{PTFE}}{1+(r_{Ag}/r_{PTFE})} \quad \text{(S1)}$$

Tab. S4. Attributions of typical C1s chemical environments. Error is expressed as one standard deviation calculated from at least three replicates.

Signal Attribution	Position (eV)
-C-C-; -C-H	284.8 ± 0.2
C-OH	286.7 ± 0.2
CF=C; C-CF; C=O	288.0 ± 0.2
-CF-	289.7 ± 0.2
$-CF_2-$	291.8 ± 0.1
$-CF_3-$	293.8 ± 0.2

Starting from the left side of the spectrum, the first three signals are unequivocally attributed to CF_3, CF_2, and CF functional groups of the polymeric backbone, respectively [3]. Signals located at lower BE values cannot be uniquely ascribed to single chemical environments; alcoholic, carbonyl, and fluorinated unsaturated groups being responsible for these features [3].

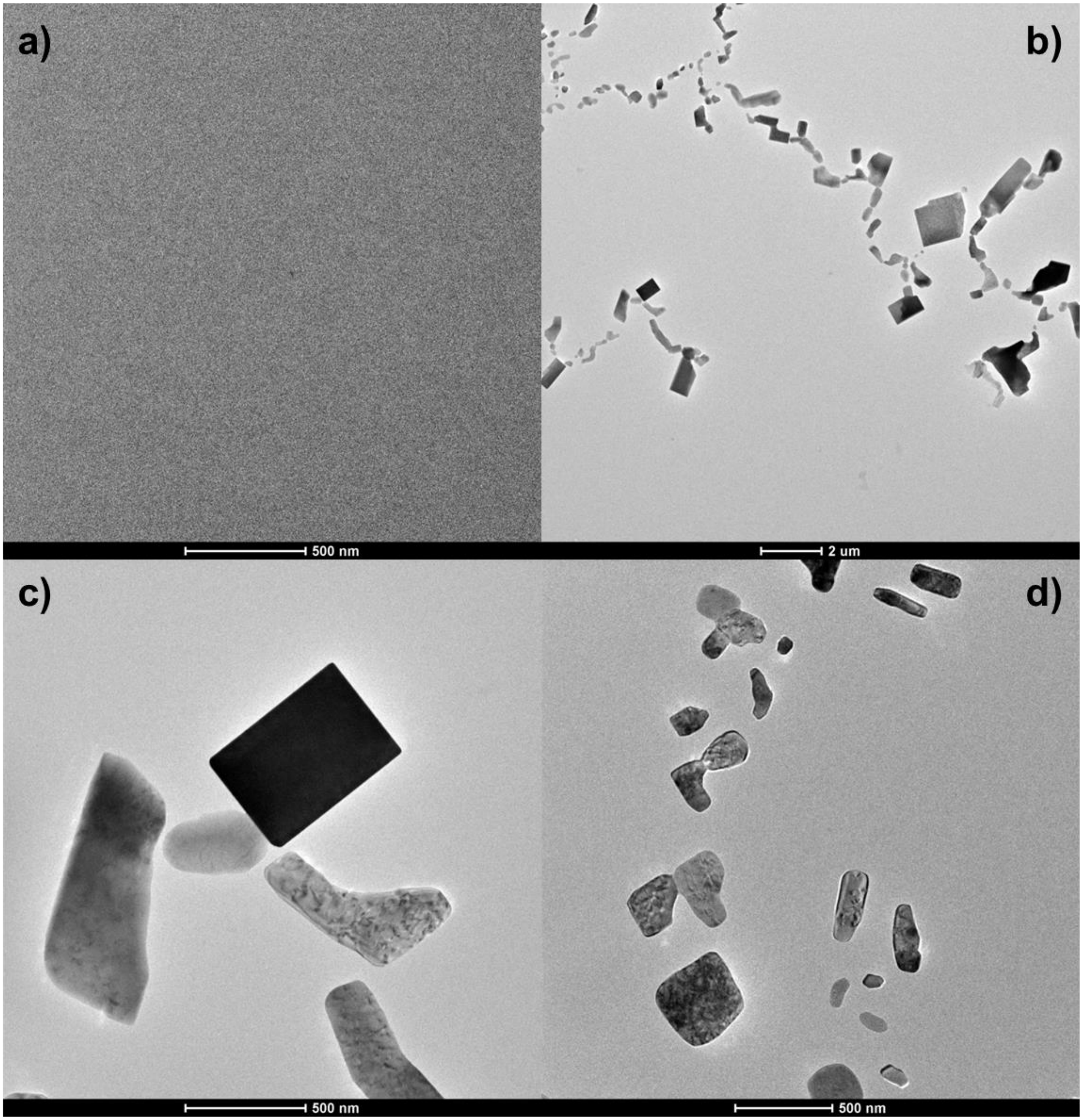

Fig. S6. TEM micrographs assessing the possible release of entire Ag nanoparticles from an Ag-CF_x composite film with φ =0.25 within 18 h of contact with an aqueous solution resembling bacteria culture medium but deprived of organic components. Most of the samples returned images totally free of any nano- or micro-phase, like in panel (a). Other fields (see panels b)-d)) were characterized by submicron crystals attributed to the inorganic electrolyte. No evidence of any spheroidal AgNPs with a size comparable to that outlined in Fig.1 could be collected.

ETAAS measurements

The experimental data were fitted using a pseudo-first-order kinetic model (S2) [4]. $[\boldsymbol{Ag}]^{\boldsymbol{0}}$ represented the amount of silver ions immediately dissolved (and therefore immediately available) in solution, $[\boldsymbol{Ag}]^{\boldsymbol{max}}$ was the maximum silver concentration in solution reached within the experiment, and $\boldsymbol{k}$ was the release kinetic constant.

$$[\boldsymbol{Ag}] = [\boldsymbol{Ag}]^{\boldsymbol{0}} + [\boldsymbol{Ag}]^{\boldsymbol{max}}\left(\boldsymbol{1} - \boldsymbol{e}^{-\boldsymbol{kt}}\right) \quad \text{(S2)}$$

Data analysis and processing was performed by SigmaPlot® 12.0 software.

Bacterial strain and culture conditions

The cultures were performed in 25 g/L Luria–Bertani broth (LB) in deionized water (Millipore Corp., Milli-Q). The LB medium was prepared from NaCl (biological grade), Tryptone (from caseine, ISO 9001 compatible), and Yeast Extract (non-selective, premium quality level, for molecular biology, ISO 9001 compatible). All these reagents were obtained from Sigma-Aldrich Chemie GmbH, Germany. Concentration of 25 g/L is relevant to the total amount of solute. For 1 L of culture broth, it is divided as follows: 10 g NaCl, 10 g of Tryptone, and 5 g of Yeast extract. Corresponding 0.5 g/L LB medium was simply obtained by 1:49 dilution of the previous one with sterilized MilliQ water. Fresh bacteria suspensions for IR-ATR measurements were obtained by inoculation of 5 mL sterile LB medium (25 g/L) in test tubes at 27 ± 1°C overnight, using a shaking incubator (KS 4000ic control, Keison Products, United Kingdom). This bacterial suspension was then used to inoculate 500 mL of sterile LB medium (25 g/L) in a 1 L Erlenmeyer flask. The resulting culture was incubated at 27 ± 1°C up to a concentration of 108 CFU/mL. The latter was evaluated following a literature procedure [5]. Cells were then harvested by centrifugation (10000 rpm, 15 minutes, 4°C) with an Eppendorf 5804/R centrifuge

Localization of ZnSe crystal IR-inactive areas

0.75 μL aliquots of a 10%w/v PSCB (Poly(styrene-co-butadiene), Sigma, 45% styrene) solution in toluene were independently deposited onto the crystal at 3 mm intervals. Prior to each deposition and spectra collection, reference spectra of air were recorded. To ensure evaporation of toluene and to obtain a stable signal, a 5-min waiting time was implemented prior to each spectrum recording. 15 spectra were recorded at 1min intervals, averaging 100 scans at 2 cm-1 spectral resolution from 4000 to 400 cm-1. Specific spectral regions were used for data evaluation (Tab. S5). Averaged integrated peak values (IPVs) of defined spectral regions at every marked position at the ATR crystal surface were then plotted versus the distance from the in-coupling facet of the ZnSe crystal (see Fig. S7).

Tab. S5. Spectral regions of interest for polystyrene-co-butadiene (PSCB). ν indicates symmetric (s), asymmetric (a) or benzene (B) stretching; δ represents aromatic/conjugated vibrational distortions.

Band	Attributions	Spectral range
■	νs (CH2), νa (CH2)	2986 - 2817 cm-1
●	νB (aromatic CH)	1613 - 1589 cm-1
▲	δ (aromatic H distortions)	1503 - 1426cm-1
▼	δ (conjugated H distortions)	1503 - 1479cm-1
♦	νB (C-C)	1460 - 1426cm-1
◄	ν'B (C-C)	1038 - 1015cm-1
►	δB (C-C)	985 - 950 cm-1
●	δ'B (C-C)	921 - 888 cm-1

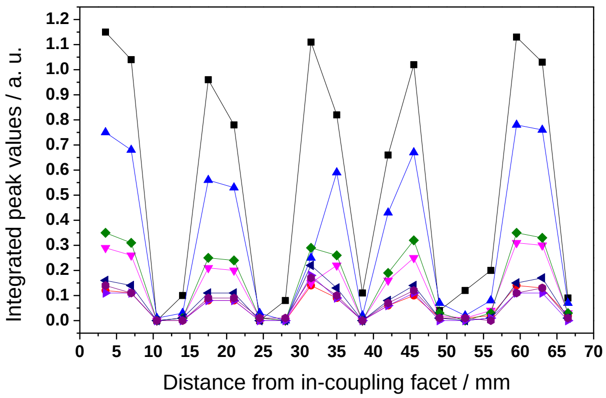

Fig. S7. Integrated peak values of polystyrene-co-butadiene (PSCB) versus distance from the in-coupling facet of the ATR crystal.

AFM experimental details

The nominal spring constant and resonance frequency of AFM tip were within the range 31-71 N/m and 160-210 kHz, respectively. To evaluate surface roughness, the root-mean-squared roughness (RMS) on bacterial surface was determined within 10x10 μm^2 areas for each sample, averaging data over 50 cells (S3):

$$\boldsymbol{RMS} = \sqrt{\frac{1}{N}\sum_{j=1}^{N} r_j^2} \quad \text{(S3).}$$

1. Dobbs, G. T. & Mizaikoff, B. Shining New Light on Old Principles: Localization of Evanescent Field Interactions at Infrared–Attenuated Total Reflection Sensing Interfaces. *Applied Spectroscopy* **60,** 573–583 (2006).
2. McWhirter, M. J., Bremer, P. J. & McQuillan, A. J. Direct Infrared Spectroscopic Evidence of pH- and Ionic Strength-Induced Changes in Distance of Attached Pseudomonas aeruginosa from ZnSe Surfaces. *Langmuir* **18,** 1904–1907 (2002).
3. Wagner, C. D. *Handbook of x-ray photoelectron spectroscopy: a reference book of standard data for use in x-ray photoelectron spectroscopy*. (Physical Electronics Division, Perkin-Elmer Corp., 1979).
4. Hahn, A., Brandes, G., Wagener, P. & Barcikowski, S. Metal ion release kinetics from nanoparticle silicone composites. *J. Controlled Release* **154,** 164–170 (2011).
5. Quilès, F., Humbert, F. & Delille, A. Analysis of changes in attenuated total reflection FTIR fingerprints of Pseudomonas fluorescens from planktonic state to nascent biofilm state. *Spectrochim. Acta, Part A* **75,** 610–616 (2010).
